# LTBP2 is a prognostic marker in head and neck squamous cell carcinoma

**DOI:** 10.18632/oncotarget.8855

**Published:** 2016-04-20

**Authors:** Liang Han, Ming Ming Tang, Xinjiang Xu, Bin Jiang, Jianfei Huang, Xingmei Feng, Jianfeng Qiang

**Affiliations:** ^1^ Department of Head and Neck Surgery, Affiliated Tumor Hospital of Nantong University, Nantong Tumor Hospital, Nantong, Jiangsu, China; ^2^ Department of Clinical Pathology, Affiliated Hospital of Nantong University, Nantong, Jiangsu, China; ^3^ Department of Stomatology, Affiliated Hospital of Nantong University, Nantong, Jiangsu, China; ^4^ Department of Graduate, Medical School of Nantong University, Nantong, Jiangsu, China

**Keywords:** head and neck squamous cell carcinoma, qPCR, immunohistochemistry, LTBP2, prognosis

## Abstract

Latent transforming growth factor (TGF)-beta binding protein 2 (LTBP2) belongs to the fibrillin/LTBP extracellular matrix glycoprotein superfamily. It plays vital roles in tumorigenesis through regulating TGFβ activity, elastogenesis and maintenance of the extracellular matrix (ECM) structure. In this study, we determined the expression levels of LTBP2 mRNA and protein in head and neck squamous cell carcinoma (HNSCC) tissues and adjacent normal tissues by quantitative reverse transcription PCR (qRT-PCR) and tissue microarray immunohistochemistry analysis (TMA-IHC) respectively. LTBP2 protein levels in cancer tissues were correlated with HNSCC patients' clinical characteristics and overall survival. Both LTBP2 mRNA and protein levels were significantly higher in HNSCC tissues than in adjacent normal tissues. High LTBP2 protein level was associated with lymph node metastasis and higher pTNM stages. High LTBP2 protein level is an independent prognostic marker in HNSCC. Our data suggest that LTBP2 acts as an oncogene in HNSCC development and progression. Detection of LTBP2 expression could be a useful prognosis marker and targeting LTBP2 may represent a novel strategy for cancer treatment through regulating activities of TGFβ.

## INTRODUCTION

Head and neck squamous cell carcinoma (HNSCC) refers to cancers arising from a variety sites within the head and neck region, including following five basic areas: the oral cavity, the pharynx, the larynx, the nasal cavity including paranasal sinuses, and the salivary glands. Overall, it accounts for approximately 600,000 new cases and 300,000 death each year worldwide [1–2]. The common risk factors include alcohol and tobacco consumption, human papillomavirus (HPV) and Epstein-Barr virus infection [3]. Males are more likely to be affected than females. The variation of disease prevalence in different regions of the world reflexes the variation of these risk factors. For example, nasopharyngeal cancer is more common in southern region of China because of EBV infection [4], while mouth and tongue cancers are more common in the subcontinent of India because of tobacco chewing [5–6]. HNSCC is a heterogeneous disease with different prognosis by disease location, with worst survival for hypopharynx and best survival for larynx. Over 50% of HNSCC cases are diagnosed as regional advanced or metastatic diseases, prognosis is better in female patients than in male patients [7–8]. Despite our improved understanding of epidemiology of HNSCC, the prognosis and overall survival of advanced HNSCC cases remain poor.

The latent transforming growth factor binding proteins (LTBPs) belong to the fibrillin superfamily of extracellular matrix (ECM) proteins characterized by a repeated domain structure including both calcium binding (cb) EGF-like domain repeats and 8-Cys TGFβ binding domain (TB domain) repeats [9]. They were originally isolated from the large latent TGFβ complex that were covalently linked to the TGFβ propeptide (latency associated peptide or LAP) via disulfide bonds, but are also associated with fibrillin microfibrils in the extracellular matrix (ECM) [9–10] In humans, there are four LTBP isoforms (LTBP1-4) and expressed in a wide variety of tissue types [11]. They are not only key regulators of biological activities of TGFβ family growth factors, but also important for the structural integrity of the ECM. Because both TGFβ family growth factors and ECM play important roles in tumorigenesis, LTBPs have also been implicated in malignant transformation.

Unlike other LTBPs, LTBP2 does not form covalent complexes with latent TGFβ. It has been hypothesized that LTBP2 indirectly regulates the activation of TGFβ by competing with LTBP1 for the same binding site to fibrillin-1 in microfibrils. In addition, LTBP2 contains an RGD integrin recognition site, thus plays an important role in cell adhesion [12]. Both tumor suppressing and tumor promoting roles have been assigned to LTBP2: it is downregulated in nasopharyngeal carcinoma (NPC) and esophageal squamous cell carcinoma (ESCC) [13–14], while it is upregulated in cervical, liver and ovarian cancers [15–17], and LTBP2 amplification has been reported in HNSCC [18].

In the current study, we determined both mRNA and protein expression of LTBP2 in HNSCC tissue samples by quantitative reverse transcription PCR (qRT-PCR) and tissue microarray immunohistochemistry analysis (TMA- IHC) respectively, and correlated to patients' clinical characteristics.

## RESULTS

### LTBP2 mRNA level was significantly higher in HNSCC tissues than in adjacent normal tissues

**Figure 1:**
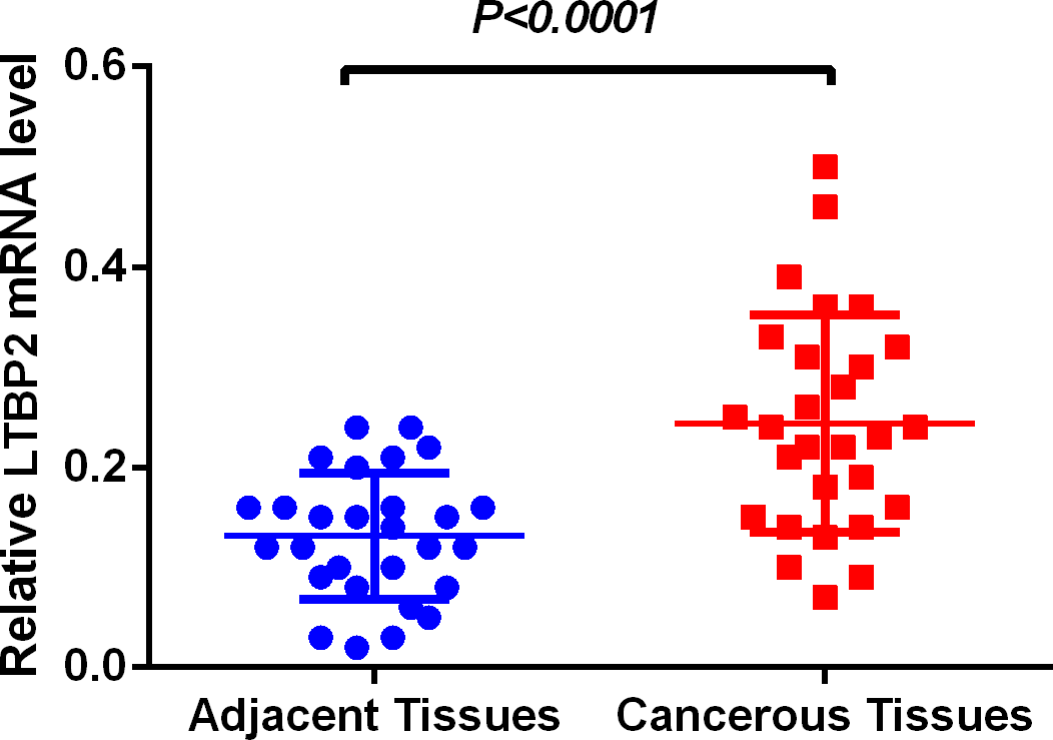
LTBP2 mRNA level was significantly higher in HNSCC tissues than in adjacent normal tissues LTBP2 mRNA was determined by qRT-PCR and relative quantification analysis by normalizing to GAPDH mRNA.

LTBP2 mRNA level was determined in 56 fresh frozen tissue samples, including 28 cancerous tissue samples and 28 matched adjacent normal tissues. Relative LTBP2 mRNA expression level was normalized to the expression of housekeeping gene GAPDH. LTBP2 mRNA expression level was significantly higher in cancerous tissues (0.2430.02049) than in adjacent normal tissues (0.13110.01190) (*P* < 0.001) (Figure 1).

### LTBP2 protein level was significantly higher in HNSCC tissues than in adjacent normal tissues

**Figure 2:**
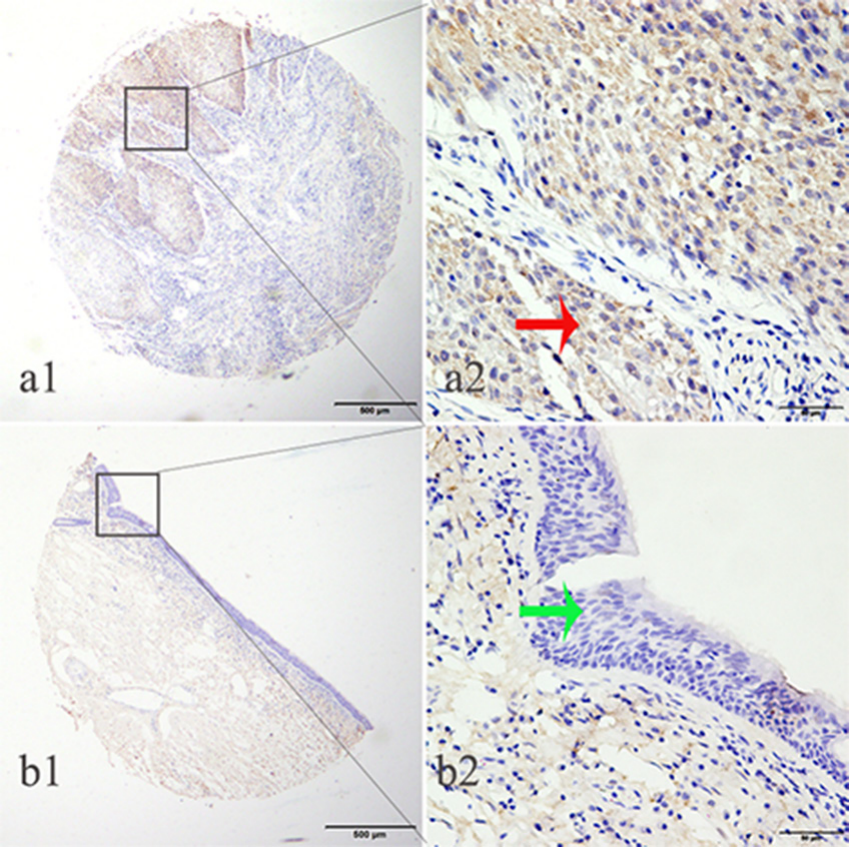
LTBP2 protein was detected in HNSCC tissues but not in adjacent normal tissues LTBP2 protein was determined by TMA-IHC, a1–a2) HNSCC tissue, positive for LTBP2 protein expression; b1–b2) adjacent normal tissue, negative for LTBP2 protein expression. a1 and b1 are ×40 magnification (bar = 500 μm), a2 and b2 are ×400 magnification (bar = 50 μm). Red arrows indicate positive LTBP2 protein expression on cancerous epithelial cytoplasm, and green arrows indicate negative LTBP2 protein expression on adjacent normal tissue.

We determined LTBP2 protein expression in 459 archived HNSCC tissue blocks, including 119 tongue squamous cell carcinoma (TSCC) tissues and 51 matched adjacent normal tissues, 87 buccal squamous cell carcinoma (BSCC) tissues and 38 matched adjacent normal tissues, 114 laryngeal squamous cell carcinoma (LSCC) tissues and 50 matched adjacent normal tissues. High LTBP2 expression was detected in 52.1% of TSCC tissues, significantly higher than 19.6% detected in adjacent normal tissues (Pearson χ^2^ = 15.438, *P* < 0.001); high LTBP2 expression was detected in 58.6% of BSCC tissues, significantly higher than 26.3% detected in adjacent normal tissues (Pearson χ^2^ = 11.047, *P* = 0.001); high LTBP2 expression was detected in 50.9% of LSCC tissues, significantly higher than 20.0% detected in adjacent normal tissues (Pearson χ^2^=13.653, *P* < 0.001) (Table 1) (Figure 2).

**Table 1:** LTBP2 protein expression in TSCC, BSCC and LSCC tissues and their adjacent normal tissues

Groups	No.	LTBP2 expression		χ^2^	*p* value
		High expression (%)	Low expression (%)		
TSCC	119	62 (52.1)	57 (47.9)	15.438	< 0.001^*^
Nomal	51	10 (19.6)	41 (80.4)		
BSCC	87	51 (58.6)	36 (41.4)	11.047	0.001^*^
Nomal	38	10 (26.3)	28 (73.7)		
LSCC	114	58 (50.9)	56 (49.1)	13.653	< 0.001^*^
Nomal	50	10 (20.0)	40 (80.0)		

* *p* < 0.05.

### Association of LTBP2 expression with HNSCC clinical characteristics

Next, we correlated LTBP2 protein expression with HNSCC patients' clinical characteristics, including tobacco and alcohol consumption. High LTBP2 protein expression was significantly associated with the presence of lymph node metastasis (*P* = 0.004) and higher stage (pTNM stage III–IV, *P* = 0.002) (Table 2).

**Table 2:** Correlation of LTBP2 protein expression with clinical characteristics of HNSCC patients

Groups	No.	LTBP2 expression		χ^2^	*p* value
		High expression (%)	No or Low expression (%)		
Total	320	171	149	-	-
Age (years)					
≤ 60 y	123	63 (51.2)	60 (48.8)	0.395	0.530
> 60 y	197	108 (54.8)	89 (45.2)		
Gender					
Female	117	64 (54.7)	53 (45.3)	0.118	0.731
Male	203	107 (52.7)	96 (47.3)		
Tobacco consumption					
Yes	65	32 (49.2)	33(50.8)	0.965	0.326
No	192	108 (56.3)	84(43.8)		
Unknown	63				
Alcohol consumption					
Yes	129	67 (51.9)	62 (48.1)	0.202	0.653
No	139	76 (54.7)	63 (45.3)		
Unknown	52				
Tumor location					
Oral	206	113 (54.9)	93 (45.1)	0.467	0.495
Larynx	114	58 (50.9)	56 (49.1)		
Tumor classification					
TSCC	119	62 (52.1)	57 (47.9)	1.325	0.516
BSCC	87	51 (58.6)	36 (41.4)		
LSCC	114	58 (50.9)	56 (49.1)		
Histopathological grade					
High	168	87 (51.8)	81 (48.2)	1.203	0.548
Moderate	126	71 (56.3)	55 (43.7)		
Low	14	9 (64.3)	5 (35.7)		
Unknown	12				
T stage					
T1 + T2	228	124 (54.4)	104 (45.6)	0.652	0.419
T3 + T4	34	21 (61.8)	13 (38.2)		
Unknown	58				
Lymph node metastasis					
Yes	74	52 (70.3)	22 (29.7)	8.293	0.004^*^
No	210	105 (50.0)	105 (50.0)		
Unknown	36				
pTNM stage					
Stage I + II	170	82 (48.2)	88 (51.8)	9.898	0.002^*^
Stage III + IV	92	63 (68.5)	29 (31.5)		
Unknown	58				

* *p* < 0.05.

### High LTBP2 expression predicts poor overall survival in HNSCC patients

**Figure 3:**
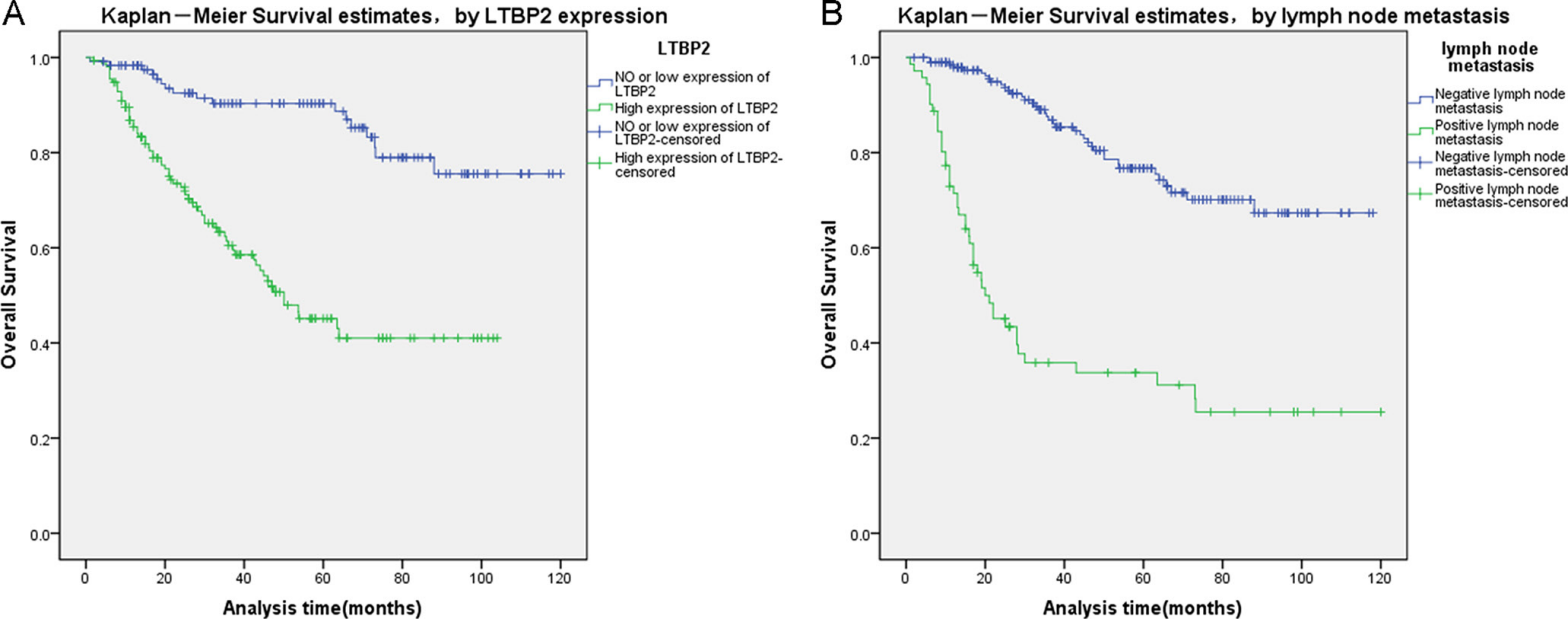
Survival curves of HNSCC patients by the Kaplan–Meier method and the log-rank test (**A**) LTBP2+ HNSCC patients (green line, 1) had significantly worse overall survival than LTBP- patients (blue line, 0). (**B**) HNSCC patients with lymph node metastasis (green line, 1) had significantly worse overall survival than patients without lymph node metastasis (blue line, 0).

Finally, we analyzed prognostic factors in HNSCC patients using both univariate and multivariate analysis. In univariate analysis, high LTBP2 expression (HR, 4.602, 95% CI: 2.686–7.883; *P* < 0.001), older age at diagnosis (HR, 1.657, 95% CI: 1.044–2.630; *P* = 0.032), T stage (HR, 2.047, 95% CI: 1.227–3.414; *P* = 0.006), histopathological grade (HR, 1.583, 95% CI: 1.129–2.218; *P* = 0.008), lymph node metastasis (HR, 5.399, 95% CI: 3.508–8.309; *P* < 0.001), and pTNM stage (HR, 4.842, 95% CI: 3.097–7.571; *P* < 0.001) were all significantly associated with overall survival. All these significant factors were then included in the multivariate analysis. In multivariate analysis, high LTBP2 expression (HR, 3.904, 95% CI: 2.253–6.766; *P* < 0.001) and presence of lymph node metastasis (HR, 2.701, 95% CI: 1.243–5.867; *P* = 0.012) remain significantly associated with poor overall survival (Table 3). Similar results were shown by the Kaplan-Meier survival curve (log rank, *P* < 0.001, Figure 3).

**Table 3:** Univariate and multivariate analysis of prognostic factors for overall survival in HNSCC Patients

	Univariate analysis			Multivariate analysis		
	HR	*p* value	95% CI	HR	*p* value	95% CI
LTBP2 expression						
High vs Low	4.602	0.001^*^	2.686–7.883	3.904	0.001^*^	2.253–6.766
Age (years)						
≤ 60 y vs > 60 y	1.657	0.032^*^	1.044–2.630			
Gender						
Female vs male	1.222	0.391	0.774–1.929			
Tobacco consumption						
Yes vs No	1.033	0.895	0.634–1.685			
Alcohol consumption						
Yes vs No	0.987	0.954	0.631–1.544			
Tumor location						
Oral vs Larynx	1.194	0.424	0.773–1.843			
Histopathological grade						
High vs Moderate vs Low	1.583	0.008^*^	1.129–2.218	1.216	0.296	0.842–1.757
T stage						
T1 + T2 vs T3 + T4	2.047	0.006^*^	1.227–3.414	1.338	0.425	0.654–2.738
Lymph node metastasis						
Yes vs No	5.399	0.001^*^	3.508–8.309	2.701	0.012^*^	1.243–5.867
pTNM stage						
Stage I, II vs Stage III, IV	4.842	0.001^*^	3.097–7.571	1.991	0.122	0.832–4.767

* *p* < 0.05.

## DISCUSSION

In the current study, we determined mRNA and protein expression levels of LTBP2 in both HNSCC and adjacent normal tissues. LTBP2 mRNA level was significantly higher in HNSCC tissues than in adjacent normal tissues. Similarly, LTBP2 protein level was significantly higher in HNSCC tissues than in adjacent normal tissues. High LTBP2 protein level was associated with lymph node metastasis and higher pTNM stages. Finally, high LTBP2 protein expression is an independent prognostic marker for poor overall survival in HNSCC patients.

LTBPs are key regulators of TGFβ activities, including cell growth, cell invasion, differentiation and morphogenesis [11]. TGFβ is secreted as a large latent complex (LLC) comprised of mature dimeric TGFβ, TGFβ propeptide (also known as latency-associated propeptide, LAP) and LTBP. LTBPs participate and regulate every step of TGFβ's biology: from folding, assembling, secretion, localization to activation. In the endoplasmic reticulum (ER), LTBP functions as the chaperone assisting the proper folding of TGFβ and LAP, assembling of TGFβ and LAP into small latent complex (SLC) then LLC, and efficient secretion of LLC [10]. Secreted LLC is stored in the ECM through interactions between LTBP and multiple extracellular proteins, and TGFβ activation is initiated through recognition of LTBP by integrin and enzymatic degradation of LTBP [10, 23–24]. The role of LTBPs in tumorigenesis is mainly through regulating TGFβ activities [25–26]. However, LTBPs also interact with other potent growth factors and important structural proteins of the ECM, playing vital roles in organ formation and tissue homeostasis [11]. It has been proposed that LTBPs can influence the structure of tumor stroma independent of TGFβ's activities [27].

During tumor initiation, TGFβ is tumor suppressive through its growth inhibition activity; but during tumor progression, TGFβ can promote invasion, metastasis, angiogenesis and immunosuppression [24]. Thus it is not surprising that both tumor promoting and tumor suppressing functions have been proposed for LTBP2. LTBP2 protein was upregulated in pancreatic cancer tissue samples [28] and plasma samples from hepatocellular carcinoma (HCC) patients [16]. LTBP2 was upregulated in cervical cancer cells and associated with clinical stage, tumor size, depth of stromal invasion and lymph node metastasis [16]. High LTBP2 protein expression predicts poor survival in serous ovarian carcinoma [17]. Mechanistic studies demonstrated that LTBP2 knockdown inhibited tumor proliferation and migration, and MAPK, PI3K-AKT, RTK and p53 signaling pathways were involved in cervical carcinogenesis [15]. In colon cancer, LTBP2 was upregulated in tumor stromal cells, but not in cancer epithelial cells [29]. On the other hand, LTBP2 was epigenetically silenced in several cancer types: LTBP2 is methylated in chronic lymphocytic leukemia (CLL) [30]; in melanoma, LTBP2 is epigenetically silenced to promote TGFβ mediated cell invasion [31]; in NPC, LTBP2 is methylated to inactivate NF-κB p65 protein mediated oncogenic signaling pathway [13, 32]. In ESCC, both tumor suppressing and tumor promoting functions have been observed for LTBP2: it was epigenetically downregulated in tumor tissues compared to normal tissues; however, low tumor cell LTBP2 expression predicts better overall survival [14]. Our data suggest that LTBP2 acts as an oncogene in HNSCC.

Our study has several limitations. First, our study samples were limited to Chinese population and subject to sample selection bias, so our conclusions may not be extended to other populations without further validation from future larger international studies. Second, our current study only included oral cavity squamous cell carcinoma (65%) and laryngeal squamous cell carcinoma (35%). It is known that HNSCC is a heterogeneous disease with different prognosis by tumor location. In European populations, the relative survival for patients with hypopharyngeal squamous cell carcinoma is only 25%, while the relative survival for patients with laryngeal squamous cell carcinoma is about 59%. Thus, we do not know whether our conclusions can be extended to all HNSCC sites. Third, we only included alcohol and tobacco consumption risk factors in our analysis, but not other two known risk factors for HNSCC: HPV and EBV infection. Fourth, we did not determine whether LTBP2 overexpression was due to DNA amplification. Earlier studies have reported that LTBP2 was highly amplified in HNSCC [14]. Finally, our clinical data were not complete, for example, we did not have treatment history during follow-up. Although we excluded the missing data points for the statistical analysis, future studies are needed to confirm our results.

In conclusion, our study demonstrates the involvement of LTBP2 in HNSCC and as an independent prognostic marker for HNSCC in Chinese population. Because of the essential role LTBPs play in regulating TGFβ activity and additional function in maintaining ECM structure, the elucidation of the molecular mechanisms of LTBPs will help understanding the role of TGFβ in tumorigenesis and tumor progression, and allow the development of optimal therapeutic agents that target the activity of TGFβ in cancer.

## MATERIALS AND METHODS

### Human tissue specimens and patient clinical information

A total of 348 HNSCC patients were included in the study. Twenty eight HNSCC patients were consented and enrolled before surgery, and 56 fresh tissue samples were collected and frozen at the time of surgery. In addition, 320 HNSCC patients provided 459 archived formalin-fixed paraffin-embedded (FFPE) tissue blocks. Clinical characteristics were obtained from patients' medical records. The study protocol was approved by the Human Research Ethics Committee of the Affiliated Hospital of Nantong University, Jiangsu, China.

### LTBP2 expression and statistical analysis

LTBP2 mRNA level was determined by quantitative reverse transcription PCR [19]. Relative quantification was performed using ΔΔCt method by first normalizing to housekeeping gene GAPDH mRNA level, then normalizing to the reference sample. By selecting and averaging the expression of five low stage (≤ stage II) tumor samples. The primers used are as follows: LTBP2 forward primer (5′- TTA CAA GCA GAG ACT CAC T-3′) and LTBP2 reverse primer (5′- ACA ACA GAA GAG ACC AGA T-3′), GAPDH forward primer (5′-TGC ACC ACC AAC TGC TTA GC-3′) and GAPDH reverse primer (5′-GGC ATG GAC TGT GGT CAT GAG-3′). LTBP2 protein expression in tissue blocks was determined using tissue microarray immunohistochemistry (TMA IHC) [19]. Rabbit polyclonal anti-human LTBP2 antibody was used (dilution 1:800, ab121193, Abcam, USA). The LTBP2 protein level was quantified using a two-level grading system, and the staining scores were defined as follows: 0–3, low expression; 4–9, high expression. The LTBP2 IHC data were also scored using the semi-quantitative H-score method and analyzed using the X-tile software program (The Rimm Lab at Yale University; http://medicine.yale.edu/lab/rimm/links/) [20–22]. Statistical analysis was performed as described before [19].
